# Supplementary material for: PD‐L1 blockade enhances response of pancreatic ductal adenocarcinoma to radiotherapy
Source: EMBO Mol Med. 2016 Dec 8;9(2):167–80. doi: 10.15252/emmm.201606674 (PMC5286375; doi:10.15252/emmm.201606674)
Supplement: Supplementary file 1 — Appendix [file EMMM-9-167-s001.pdf]

Appendix Figure S1

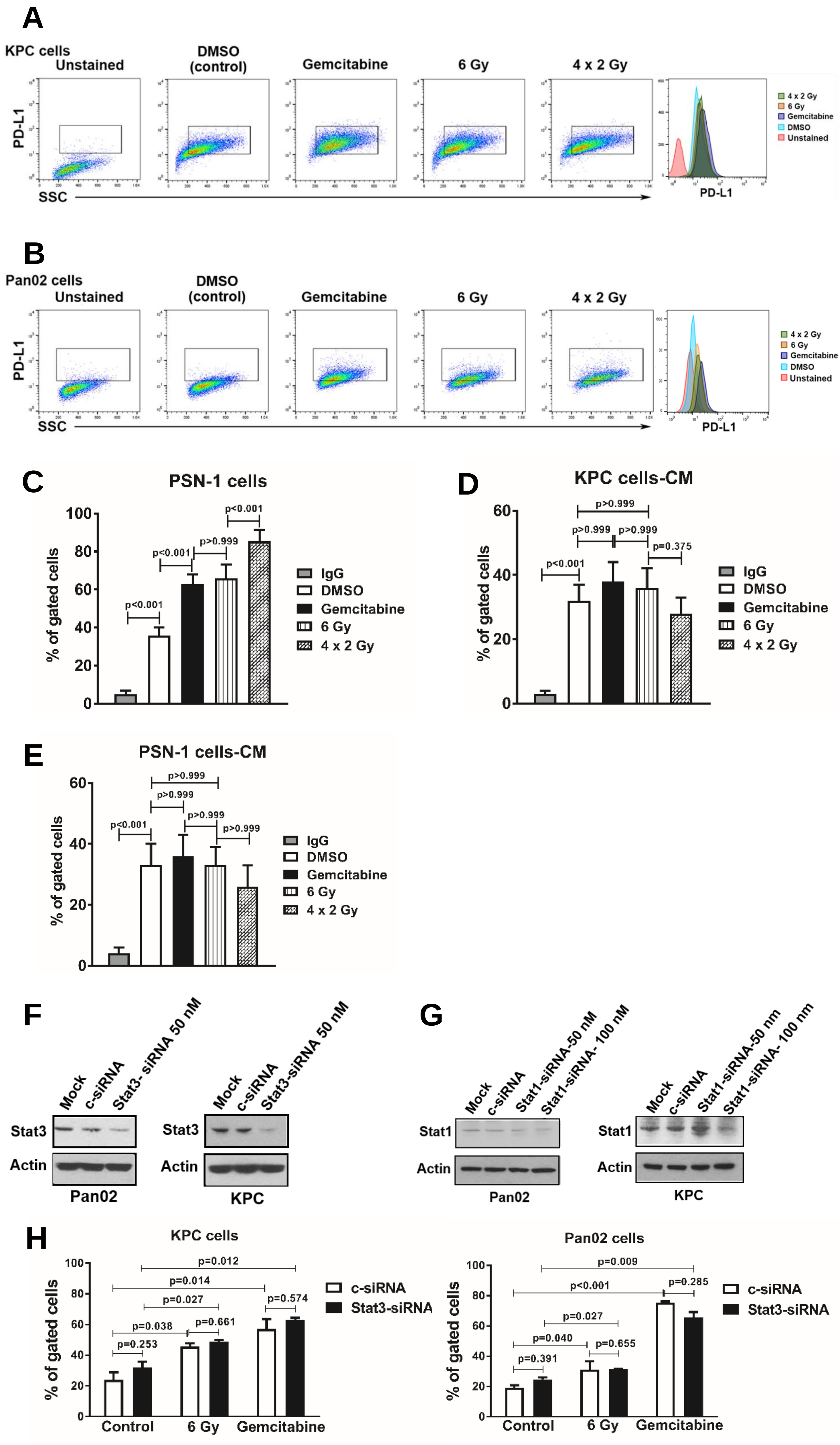

Appendix Figure S1. RT and chemotherapy alter PD-L1 expression in PDAC cells. Flow cytometric analysis of PD-L1 expression in ***(A)*** KPC and ***(B)*** Pan02 cells following treatment with gemcitabine and RT. Representative dot plots are shown. ***(C)*** Flow cytometric analysis of PD-L1 in PSN-1 human PDAC cells 24 hrs after RT and gemcitabine. Gemcitabine was diluted in DMSO (vehicle) and hence we used DMSO for the untreated control group and the RT groups as well (means ± SD, n=3, One-way ANOVA, Bonferroni test). Conditioned medium from ***(D)*** KPC and ***(E)*** PSN-1 cells pre-treated with RT and gemcitabine was collected 24 hrs later, added to treatment-naïve PDAC cells and PD-L1 expression analysed. Conditioned medium did not alter PD-L1 expression in KPC and PSN-1 cells. Flow cytometry gating for PD-L1 was performed as in main Figure 1 (means ± SD, n=3, One-way ANOVA, Bonferroni test). ***(F-G)*** Western blot confirmed blockade of Stat1 and Stat3 in KPC and Pan02 cells using siRNA (50 nM and 100nM for Stat1; 50 nM for Stat3) as indicated. ***(H)*** Flow cytometric analysis of PD-L1 after gemcitabine and RT (as described above) in KPC and Pan02 following Stat3 downregulation by siRNA (50 nM). A control siRNA (c-siRNA) was used as well (means ± SD, n=3, Student's t-test).

Supplementary Figure 2

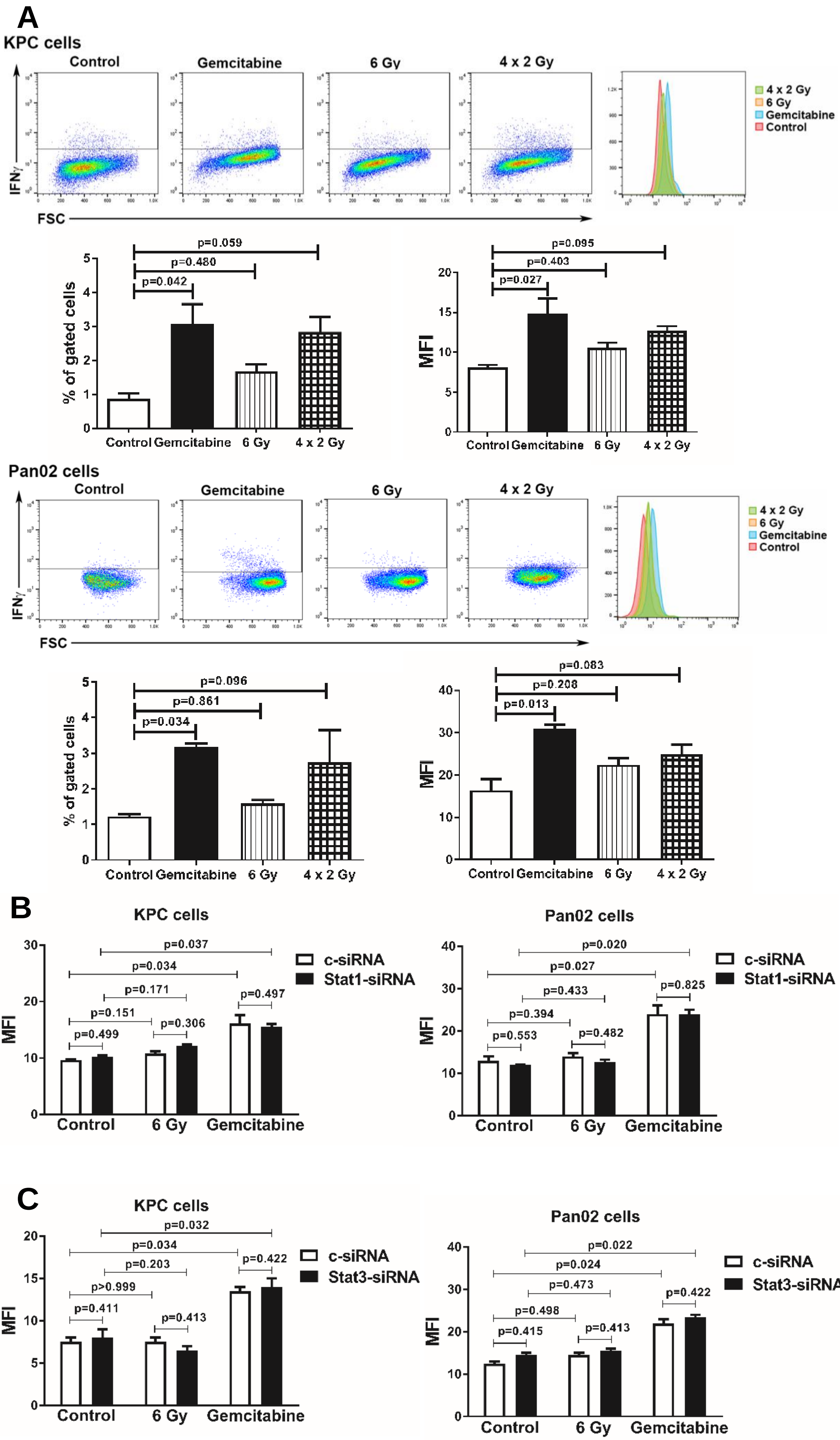

Appendix Figure S2. (A) Flow cytometric analysis of IFNγ in KPC and Pan02 cells 24 hrs after RT and gemcitabine. Gemcitabine was diluted in DMSO (vehicle) and hence we used DMSO for the untreated control group and the RT groups as well (means ± SD, n=3, One-way ANOVA, Bonferroni test). Flow cytometric analysis of IFNγ in KPC and Pan02 cells treated with gemcitabine and RT after downregulation of (B) Stat1 (50 nM and 100 nM) and (C) Stat3 by siRNA (50 nM). A control siRNA (c-siRNA) was used as well (means ± SD, n=3, Student's t-test). MFI, mean fluorescence intensity.

Appendix Figure S3

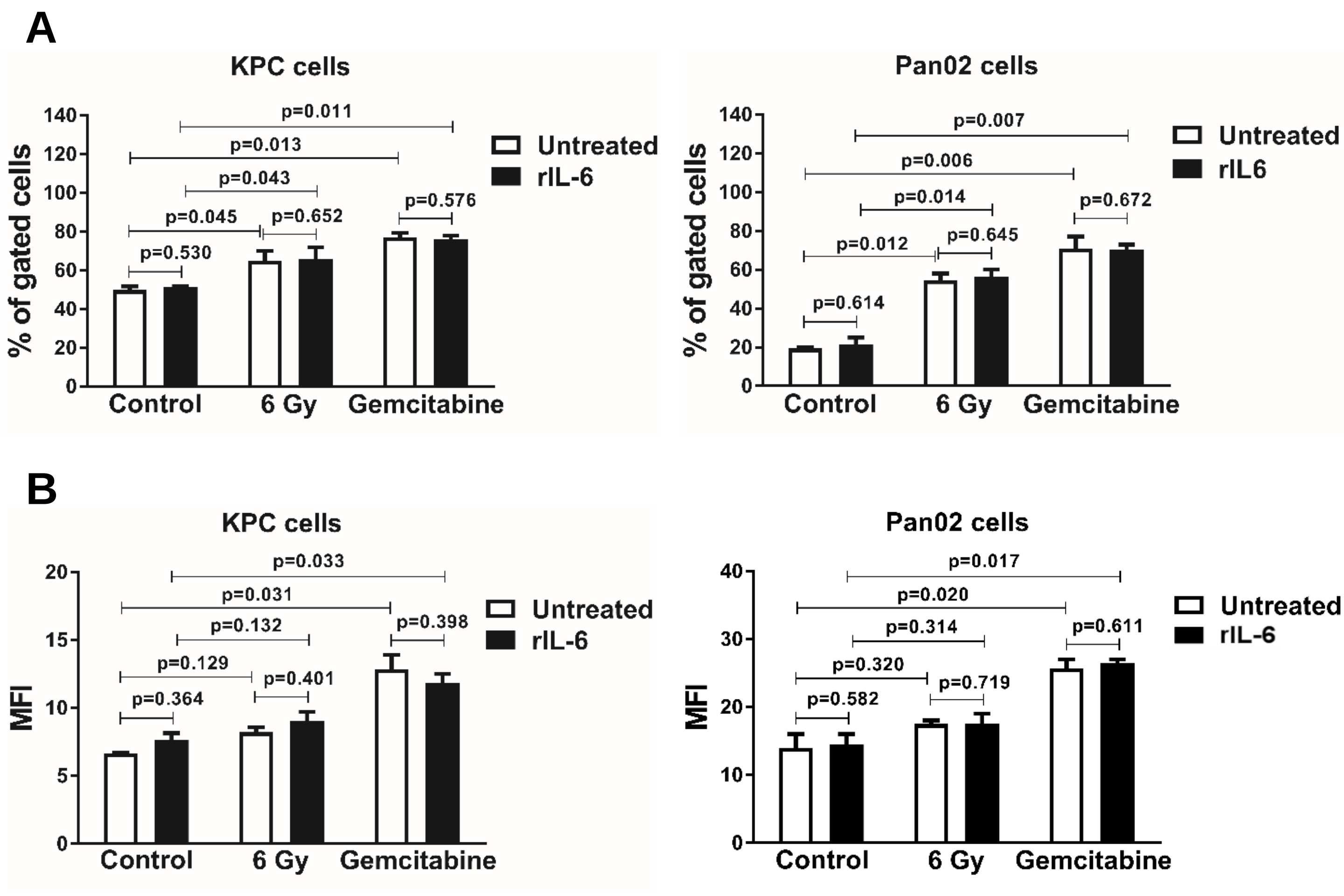

Appendix Figure S3. Flow cytometric analysis of ***(A)*** PD-L1 and ***(B)*** IFNγ upon addition of recombinant IL-6 (50 ng/ml) in KPC and Pan02 cells. Analysis was performed 24 hrs after RT and gemcitabine. Gemcitabine was diluted in DMSO (vehicle) and hence we used DMSO for the control group and the RT groups as well (means ± SD, n=3, Student's t-test). MFI, mean fluorescence intensity.

Appendix Figure S4

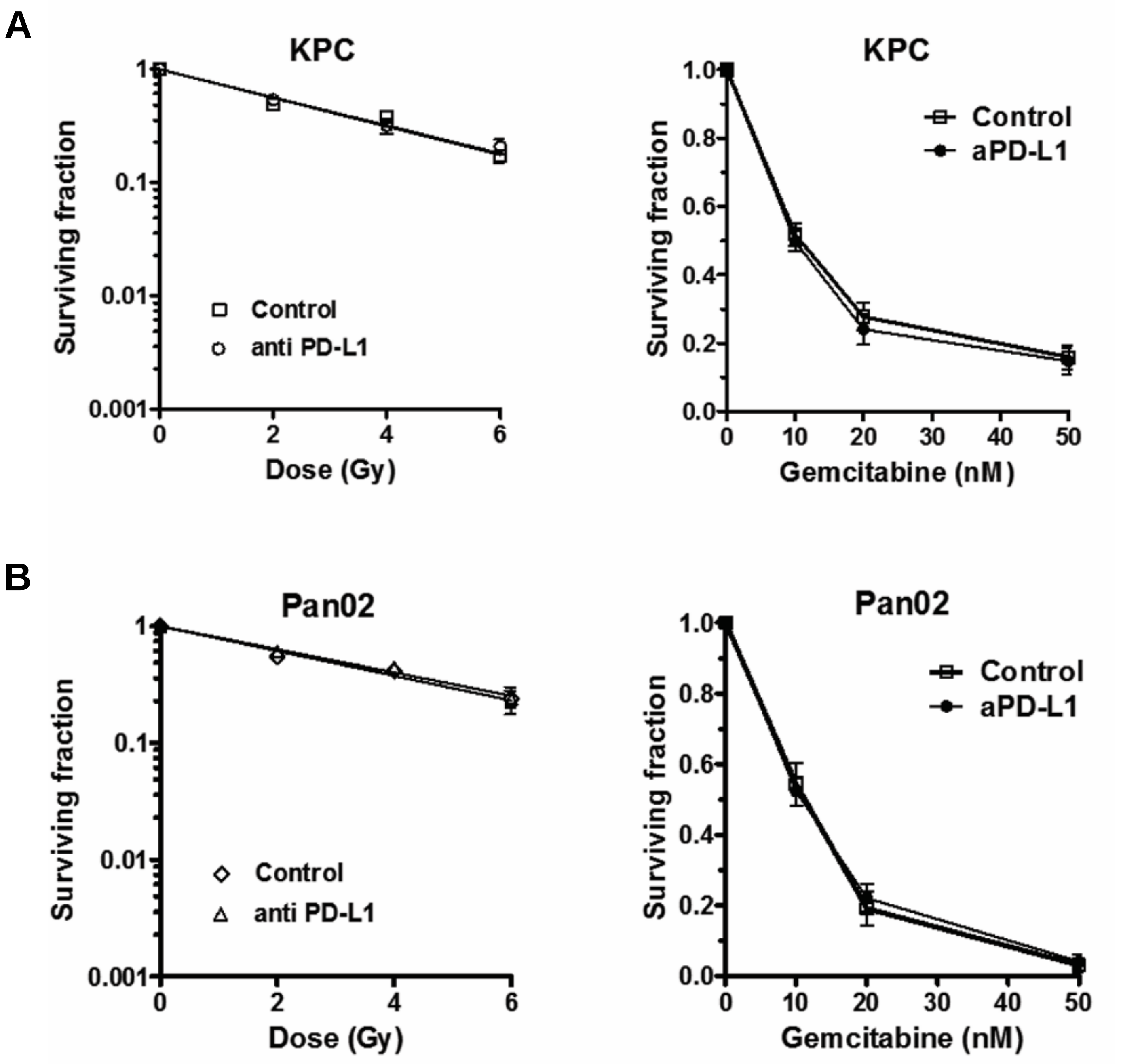

Appendix Figure S4. Clonogenic survival assay in (A) KPC cells and (B) Pan02 cells after treatment with RT and gemcitabine + anti PD-L1 (2 µg/ml), as indicated. Gemcitabine was diluted in DMSO (vehicle) and hence we used DMSO for the untreated control group as well (means ± SD, n=3, Student's t-test)

Appendix Figure S5

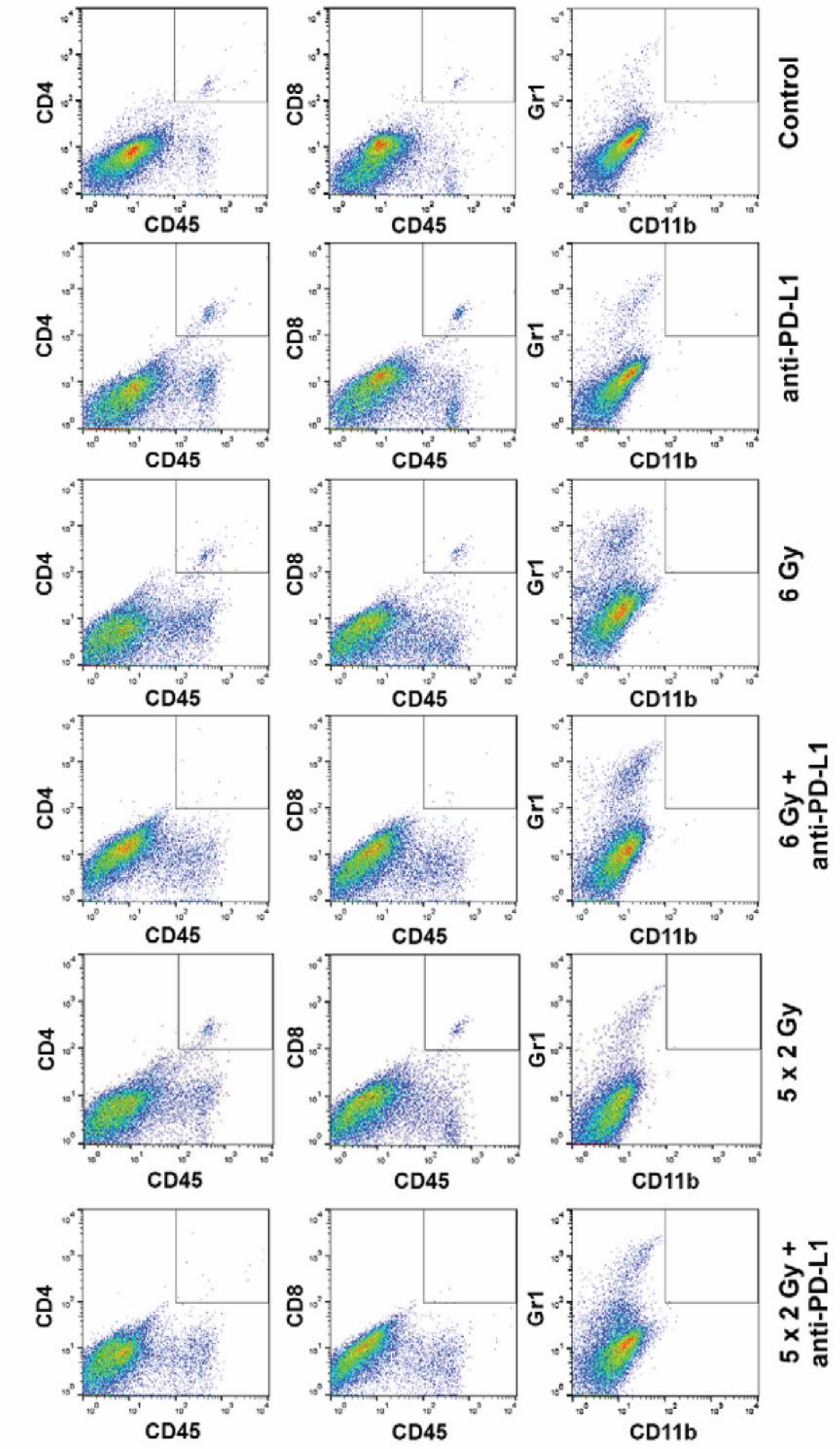

Appendix Figure S5. Flow cytometry analysis of either immune activator or immune effectors shown in main Figure 2. Representative plots are shown for the different groups as indicated.

Appendix Figure S6

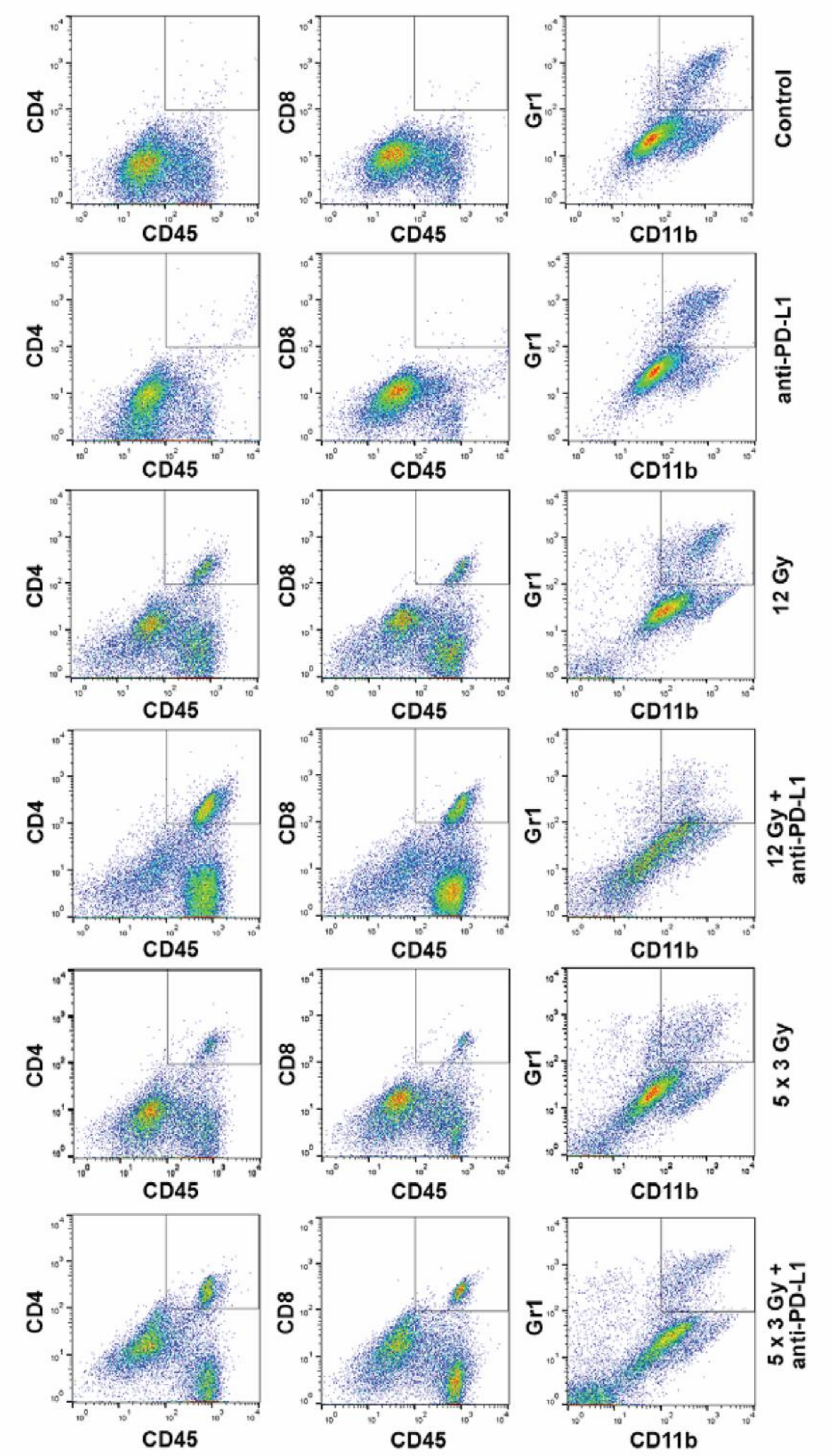

Appendix Figure S6. Flow cytometry analysis of either immune activator or immune effectors shown in main Figure 3. Representative plots are shown for the different groups as indicated.

Appendix Figure S7

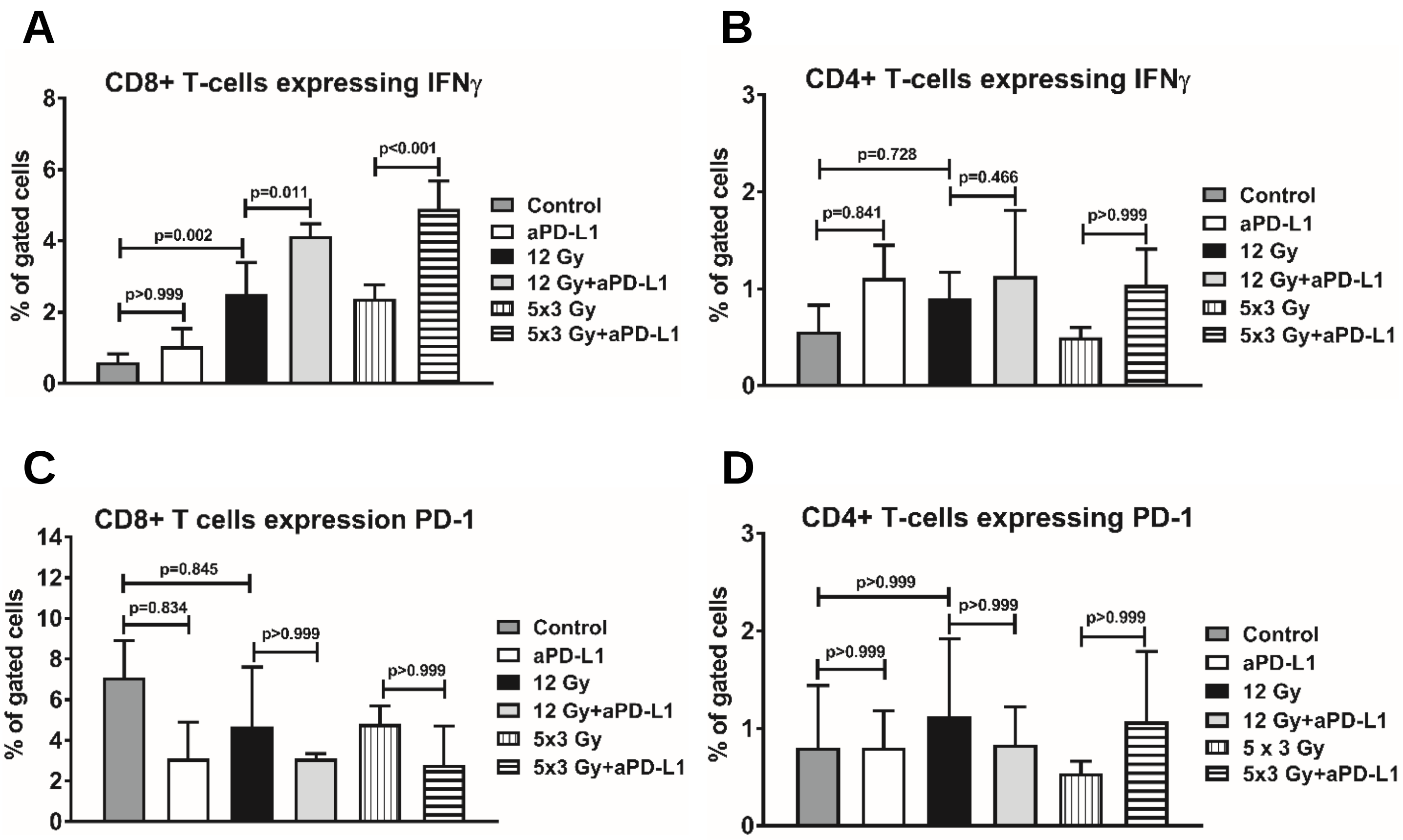

Appendix Figure S7. IFNγ expression was analysed by flow cytometry in ***(A)*** CD8+ T-cells and ***(B)*** CD4+ T-cells in blood samples of KPC tumor bearing mice from the different treatment groups, as indicated (n=5 per group; means ± SD, n=1, One-way ANOVA, Bonferroni test). PD-1 expression was also analysed in ***(C)*** CD8+ T-cells and ***(D)*** CD4+ T-cells using blood samples from the same experiment. Quantitative data are shown (n=5 per group; means ± SD, n=1, One-way ANOVA, Bonferroni test)

Appendix Figure S8

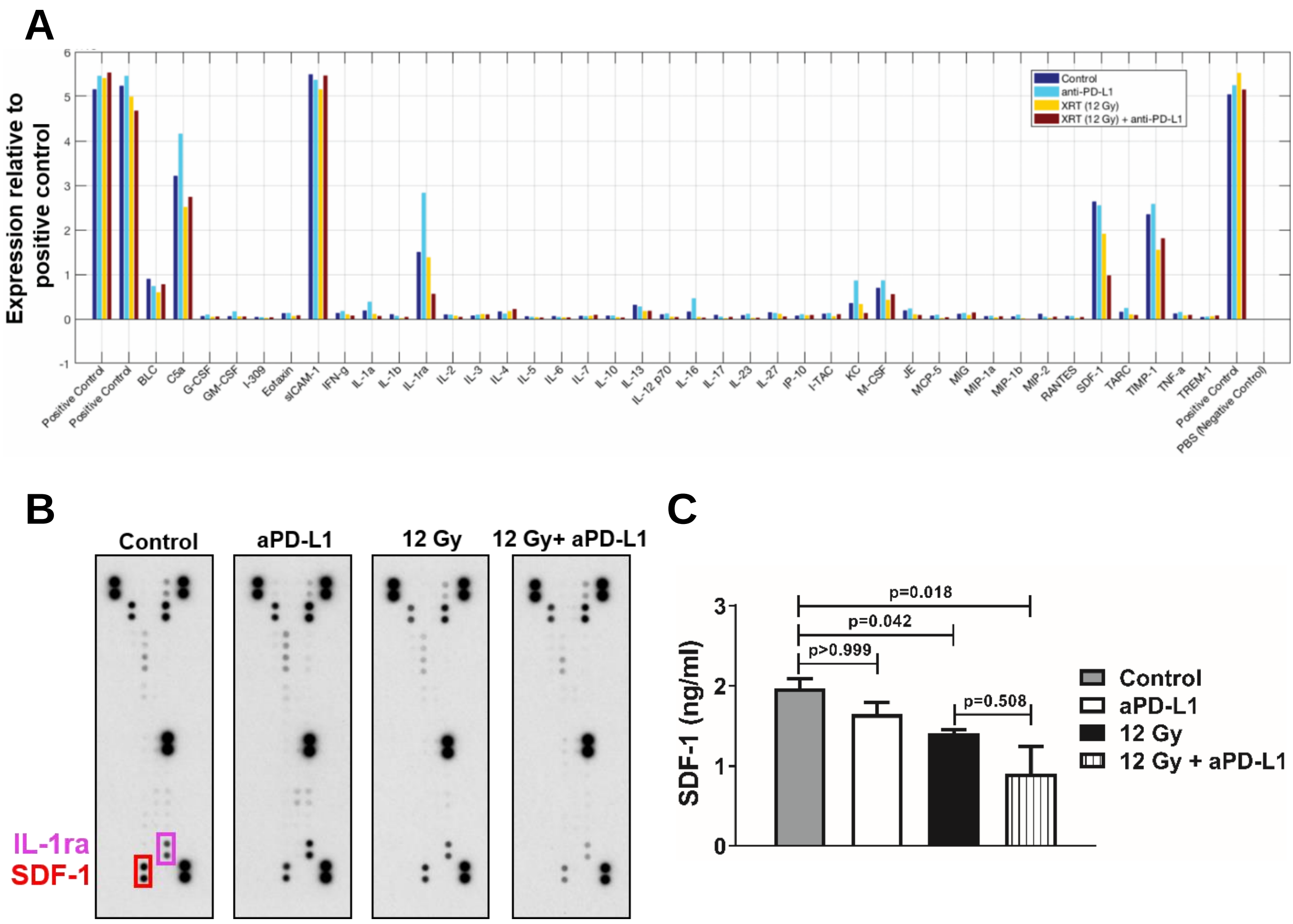

Appendix Figure S8. ***(A-B)*** Cytokine array analysis using a standard kit in blood serum of KPC allografts obtained 5 days after treatment as indicated. Stromal derived factor 1 (SDF-1) and interleukin receptor 1ra (IL-1ra) appeared downregulated in the12 Gy plus anti-PD-L1 group. ***(C)*** ELISA (n=6 mice per group) confirmed significant downregulation of SDF-1 both in the RT and the combination group (means ± SD, n=3, One-way ANOVA, Bonferroni test).

Appendix Figure S9

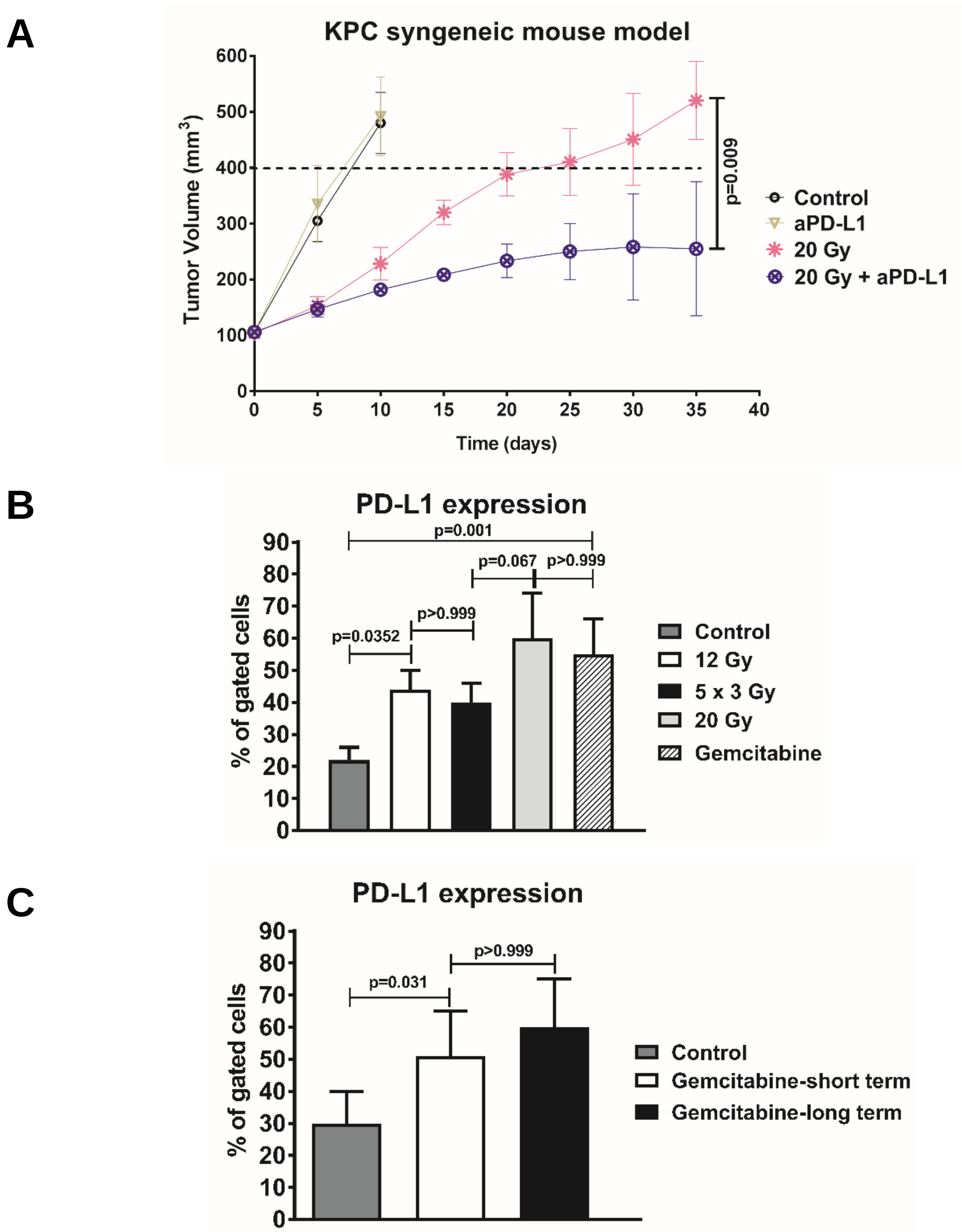

Appendix Figure S9. ***(A)*** KPC tumor growth delay after treatment with either 20 Gy (day 0), anti-PD-L1 (days 0, 3, 6 and 9) or their combination, as indicated. The radiosensitizing effect of anti-PD-L1 after 20 Gy was assessed as shown by the capped line. (n=6 mice per group; means ± SD, n=1, One-way ANOVA, Bonferroni test). No weight loss was observed in the *in vivo* experiments. ***(B)*** Expression of PD-L1 in the syngeneic KPC xenografts following treatment with RT and gemcitabine. PD-L1 expression was analysed 5 days post-treatment in the syngeneic KPC xenografts after either control (vehicle), 12 Gy (as described in Figure 4A), 5x3 Gy (as described in Figure 4B), 20 Gy (as described in Figure 4D) and gemcitabine (shown in main Figure 5A) treatment (means ± SD, n=1, One-way ANOVA, Bonferroni test). ***(C)*** Similarly, PD-L1 expression was analysed in KPC transgenic mice either short-term (24 hr) or long term (3-7 weeks) after gemcitabine (100 mg/kg i.p. days 0, 3, 6, and 9). (n=5 per group; means ± SD, n=1, One-way ANOVA, Bonferroni test).

Appendix Figure S10

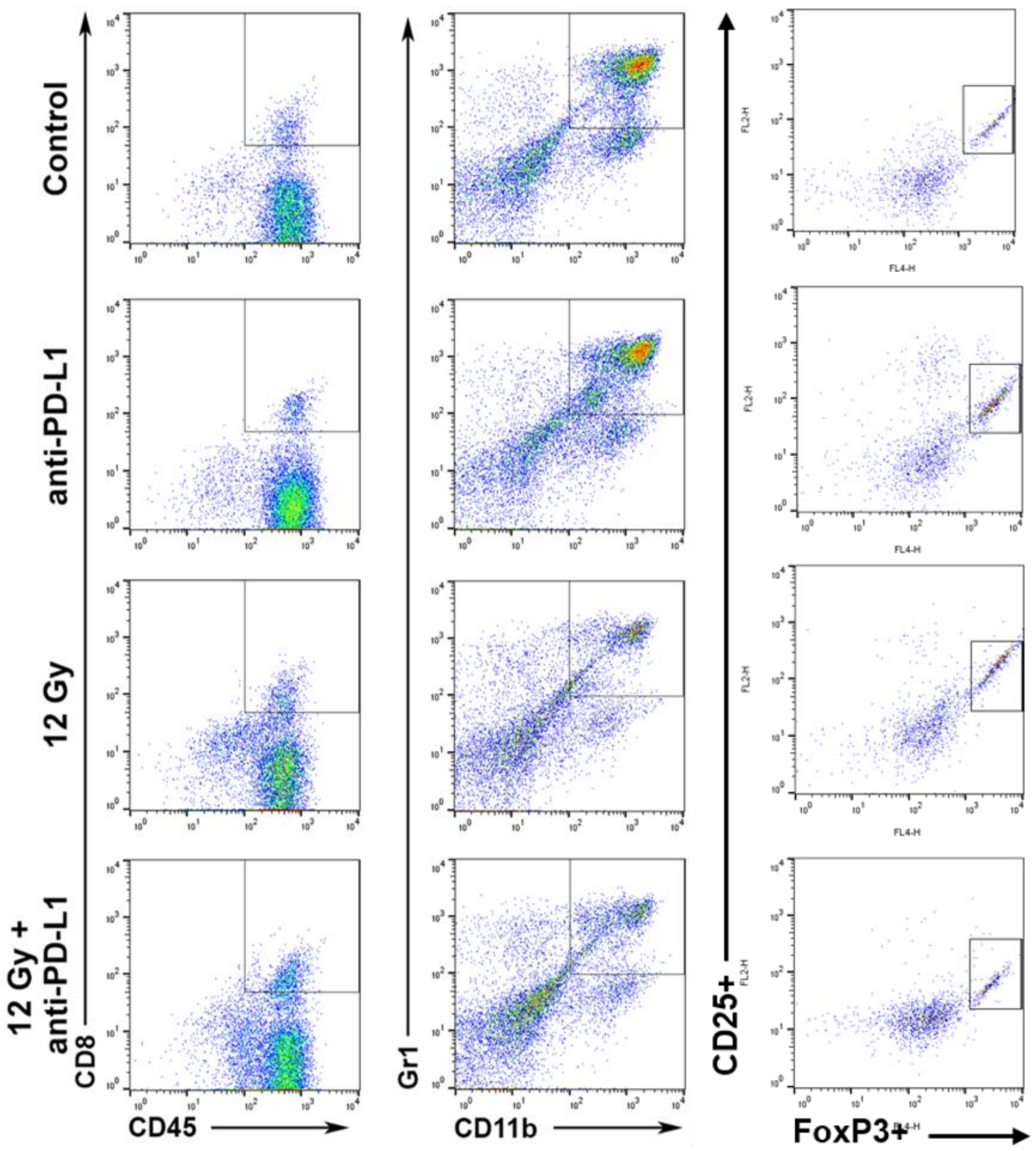

Appendix Figure S10. Flow cytometry dot plots of CD45+CD8+, CD11b+Gr1+ and CD4+CD25+FOXP3+ cell populations in the livers of the mice from the different treatment groups as shown in main Figure 6.

Appendix Figure S11

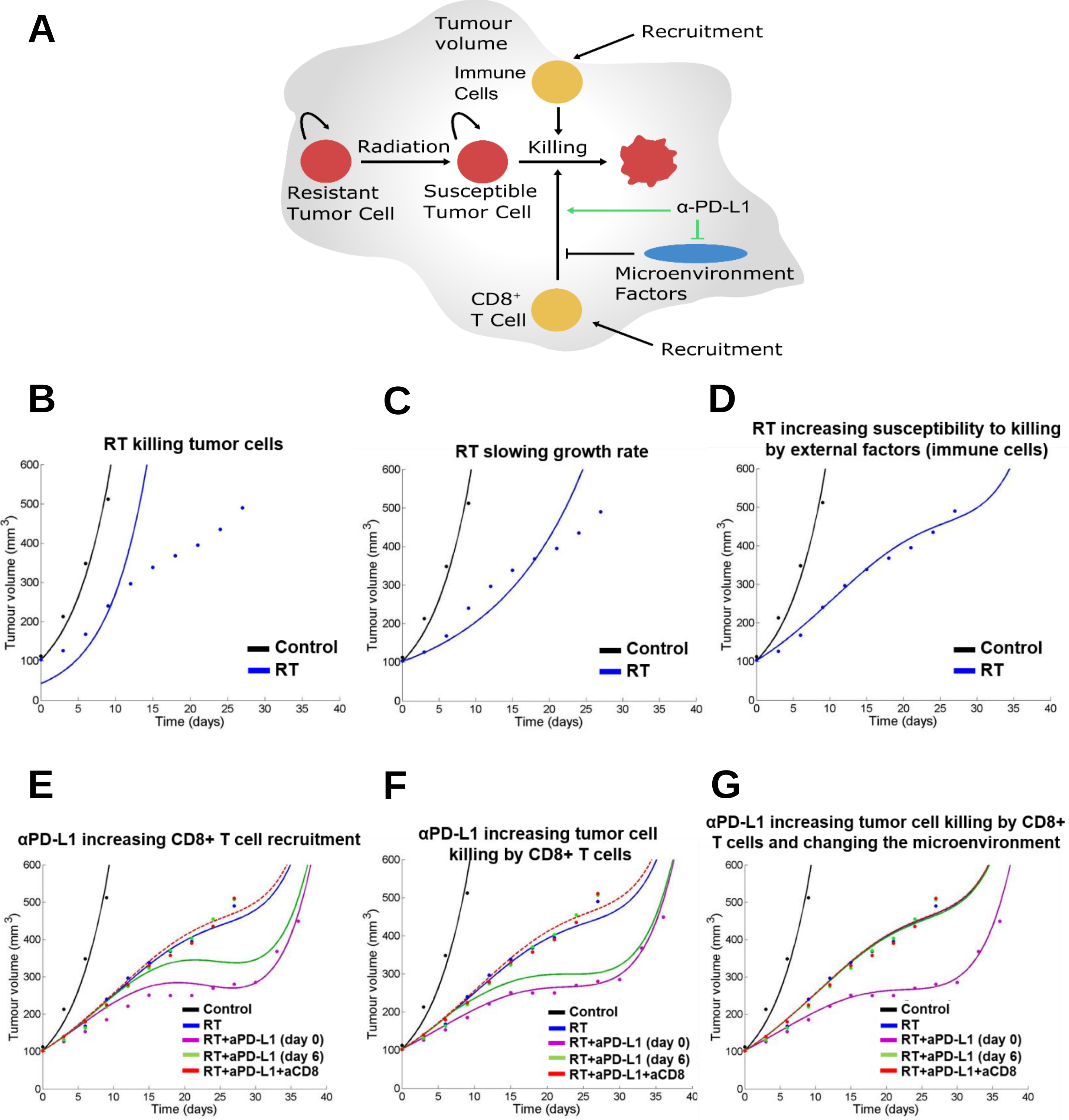

Appendix Figure S11. Mathematical model of immune cells interacting with tumor cells reproduces experimental data and provides insight into the mechanism of the synergy between radiation and anti-PD-L1. ***(A)*** Schematic of mathematical model. RT renders tumor cells susceptible to killing by immune cells. The immune cell compartment is split into CD8+ T cells and other immune cells (see main text). We include the generation of immunosuppressive microenvironment factors that reduce the ability of CD8+ T cells to kill tumor cells. We model anti-PD-L1 therapy as both reducing the formation of suppressive microenvironment factors and by directly increasing the ability of T cells to kill tumor cells. ***(B)*** Calculated tumor growth if RT kills a large fraction of tumor cells. ***(C)*** Calculated tumor growth if RT slows down the effective growth rate, i.e. increasing cell death or slowing down cell division. ***(D)*** Mathematical model calculations assuming RT converts a large fraction of tumor cells into a state susceptible to killing by immune cells. ***(E)*** Mathematical model calculations assuming PD-L1 blockade only increases CD8+ T cell-mediated tumor cell killing. ***(F)*** Mathematical model calculations assuming PD-L1 blockade only increases CD8+ T cell recruitment compared to experimental data. ***(G)*** Calculations of the mathematical model when PD-L1 blockade increases CD8+ T cell killing and reduces the suppressive microenvironment. Experimental data is shown as filled circles and mathematical model calculations are shown as solid or dashed lines.
