# Supplementary figures and images for: PD‐L1 blockade enhances response of pancreatic ductal adenocarcinoma to radiotherapy
Source: EMBO Mol Med. 2016 Dec 8;9(2):167–80. doi: 10.15252/emmm.201606674 (PMC5286375; doi:10.15252/emmm.201606674)

Source data Appendix Figure S1

F

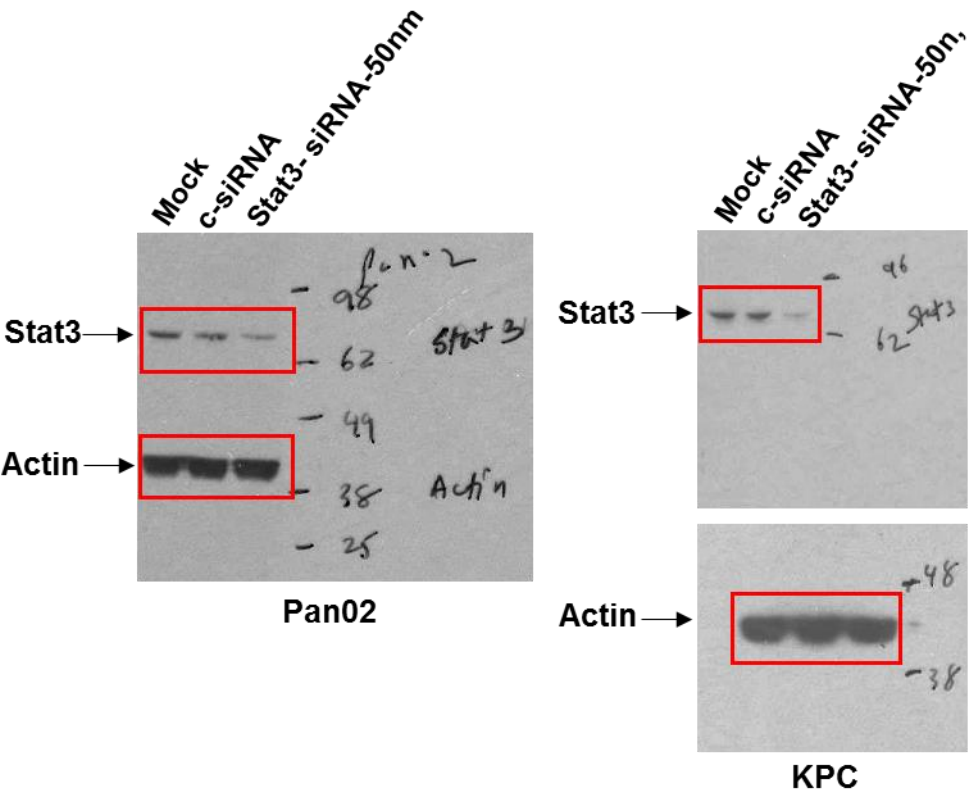

Knockdown of Stat3 by siRNA

G

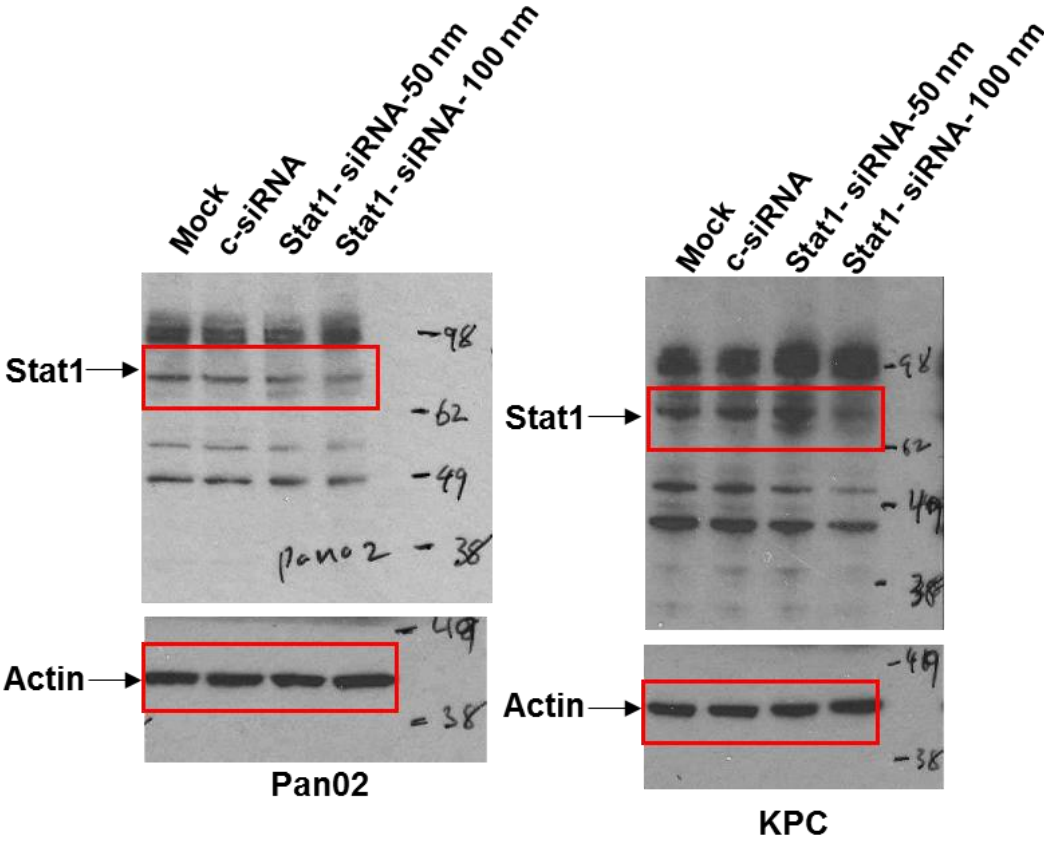

Knockdown of Stat1 by siRNA

Supplement: Supplementary file 2 — Source Data for Appendix [file EMMM-9-167-s002.zip › SourceDataAppendixFigureS1.pdf]
