# Supplementary material for: PD‐L1 blockade enhances response of pancreatic ductal adenocarcinoma to radiotherapy
Source: EMBO Mol Med. 2016 Dec 8;9(2):167–80. doi: 10.15252/emmm.201606674 (PMC5286375; doi:10.15252/emmm.201606674)
Supplement: Supplementary file 4 — Source Data for Figure 1 [file EMMM-9-167-s003.pdf]

Source data Figure 1

C

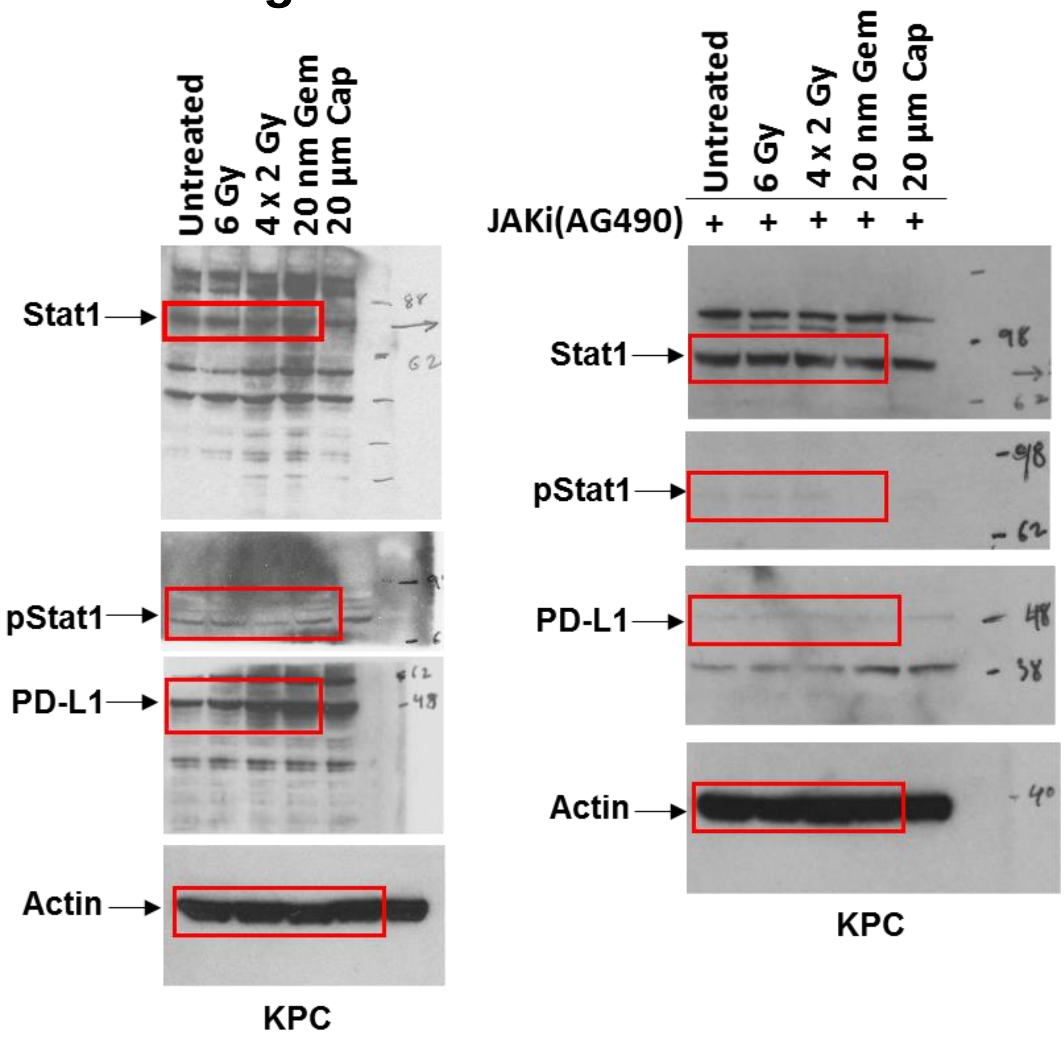

D

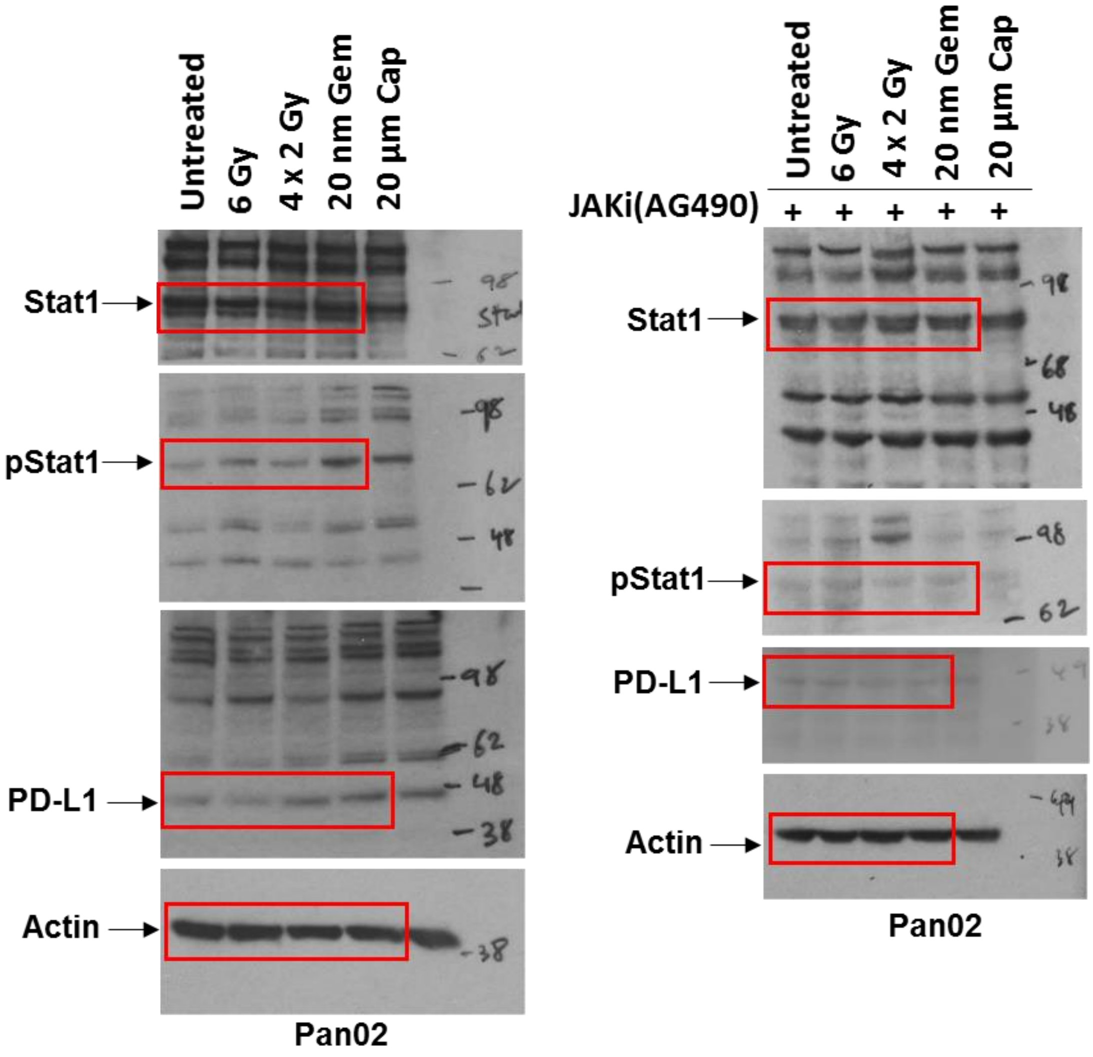

Effect of Jak/Stat inhibition on PD-L1 expression after RT (6 Gy, 4x2 Gy), gemcitabine (20nm) and capecitabine (20µm) chemotherapy. Of note, in the present work we concentrated on RT and gemcitabine chemotherapy only.
